# Comprehensive behavioral study of mGluR3 knockout mice: implication in schizophrenia related endophenotypes

**DOI:** 10.1186/1756-6606-7-31

**Published:** 2014-04-23

**Authors:** Ryuta Fujioka, Takenobu Nii, Akiko Iwaki, Atsushi Shibata, Isao Ito, Kiyoyuki Kitaichi, Masatoshi Nomura, Satoko Hattori, Keizo Takao, Tsuyoshi Miyakawa, Yasuyuki Fukumaki

**Affiliations:** 1 Division of Human Molecular Genetics, Research Center for Genetic Information, Medical Institute of Bioregulation, Fukuoka 812-8582, Japan; 2 Department of Biology, Faculty of Science, Kyushu University, Fukuoka 812-8581, Japan; 3 Department of Pharmacology, Nagasaki International University, Sasebo 859-3298, Japan; 4 Department of Pharmacology, Gifu University Hospital, Gifu 501-1194, Japan; 5 Department of Medicine and Bioregulatory Science, Graduate School of Medical Science, Kyushu University, Fukuoka 812-8582, Japan; 6 Division of Systems Medical Science, Institute for Comprehensive Medical Science, Fujita Health University, Toyoake 470-1192, Japan; 7 CREST, Japan Science and Technology Agency, Kawaguchi 332-0012, Japan; 8 Section of Behavior Patterns, Center for Genetic Analysis of Behavior, National Institute for Physiological Sciences, Okazaki 444-8787, Japan

**Keywords:** Metabotropic glutamate receptors, *Grm3*, Knockout mice, Working memory, Reference memory, Contextual memory, Hyperactivity, LTP, Microdialysis, Schizophrenia

## Abstract

**Background:**

We previously performed systematic association studies of glutamate receptor gene family members with schizophrenia, and found positive associations of polymorphisms in the *GRM3* (a gene of metabotropic glutamate receptor 3: mGluR3) with the disorder. Physiological roles of *GRM3* in brain functions and its functional roles in the pathogenesis of schizophrenia remain to be resolved.

**Results:**

We generated mGluR3 knockout (KO) mice and conducted comprehensive behavioral analyses. KO mice showed hyperactivity in the open field, light/dark transition, and 24-hour home cage monitoring tests, impaired reference memory for stressful events in the Porsolt forced swim test, impaired contextual memory in cued and contextual fear conditioning test, and impaired working memory in the T-Maze forced alternation task test. Hyperactivity and impaired working memory are known as endophenotypes of schizophrenia. We examined long-term synaptic plasticity by assessing long-term potentiation (LTP) in the CA1 region in the hippocampi of KO and wild-type (WT) mice. We observed no differences in the amplitude of LTP between the two genotypes, suggesting that mGluR3 is not essential for LTP in the CA1 region of the mouse hippocampus. As hyperactivity is typically associated with increased dopaminergic transmission, we performed *in vivo* microdialysis measurements of extracellular dopamine in the nucleus accumbens of KO and WT mice. We observed enhancements in the methamphetamine (MAP)-induced release of dopamine in KO mice.

**Conclusions:**

These results demonstrate that a disturbance in the glutamate-dopamine interaction may be involved in the pathophysiology of schizophrenia-like behavior, such as hyperactivity in mGluR3 KO mice.

## Background

Glutamate receptors play central roles in neuronal excitation in the mammalian central nervous system. Glutamate receptors have been categorized into two classes: ionotropic glutamate receptors and metabotropic glutamate receptors (mGluRs) [1]. mGluRs are G protein-coupled receptors and consist of eight different subtypes that have been subdivided into three groups (I–III) based on their sequence homology [2]. mGluR3 and mGluR2 have been classified as group II mGluRs due to their sequence homology and shared signal propagation mechanism [3-5]. mGluR2 is expressed in neuronal cells, whereas mGluR3 is distributed in both neuronal and glial cells. Group II mGluRs play an important role in synaptic plasticity, including the regulation of LTP [6]. An inhibitory effect of mGluR3 on LTP was observed in the rat dentate gyrus [7,8]. These events mainly occur in presynaptic sites, through their inhibition of glutamate release [9]. Group II mGluRs function as heteroceptors, regulating the release of other neurotransmitters, including dopamine [5,9]. Since mGluR2/3 activation leads to a net reduction in glutamate neurotransmission, the ligands for these receptors may be potential therapeutic agents for a wide range of psychiatric disorders [10]. Phencyclidine (PCP), an antagonist of the N-methyl-D-aspartate (NMDA) receptor of glutamate receptor, induces schizophrenic symptoms in humans [11]. The psychotic effect induced by PCP is attenuated by administration of agonists specific to mGluR2 and mGluR3 [12]. We, therefore, previously conducted systematic studies on the association of glutamate receptor genes with schizophrenia. We found a positive association between single nucleotide polymorphisms (SNPs) located in *GRM3* and schizophrenia in the Japanese population [13]. Four subsequent studies also showed the positive association between SNPs located in *GRM3* and schizophrenia [14-17], although negative results were reported [18-20]. Resequencing of all exons and splice sites of *GRM3* of schizophrenia patients revealed no missense or splice-site SNPs, suggesting that intronic SNPs located in *GRM3* or related haplotypes may affect subtle regulatory effects on *GRM3* transcription [14]. To explore the physiological roles of *GRM3* in brain functions and its functional roles in the pathogenesis of schizophrenia, we generated *Grm3* knockout (KO) mice and conducted comprehensive behavior tests, electrophysiological, and pharmaco-physiological analyses.

## Results

### Generation of mGluR3 KO mice

**Figure 1:**
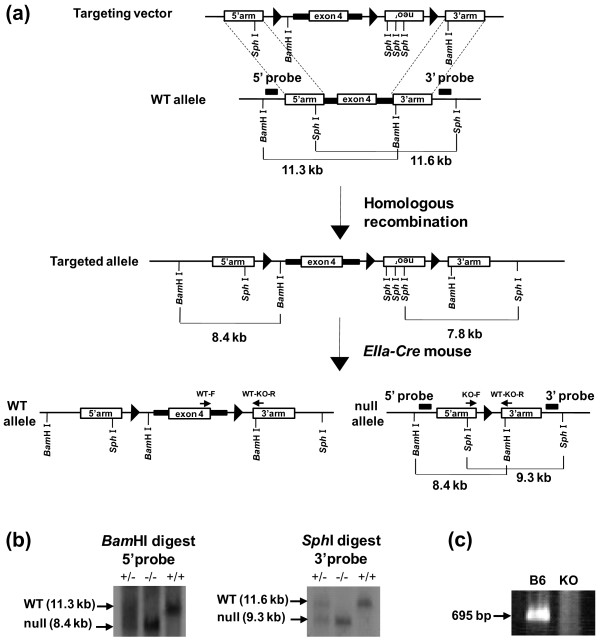
**Generation and validation of mGluR3 KO mice. (a)** WT and KO alleles: Closed boxes indicate the approximate positions of the 5′ and 3′ probes. The closed triangle indicates the *loxP* sequence. **(b)** Southern blots of *Bam*H I and *Sph* I-digested genomic DNA were hybridized with the 5′ probe and 3′ probe shown in **(a)**, respectively. The DNA bands indicated by the arrows correspond to the *Bam*H I and *Sph* I-fragments. **(c)** RT-PCR analysis of mGluR3 expression in the brains of C57BL/6J (B6) and KO mice using specific primers located in exons 3 and 4 of *Grm3*. Total RNAs were isolated from the brains of B6 and KO mice. A PCR product of 695 bp was detected in B6, but not in KO mice.

To generate mGluR3 KO mice, three fragments corresponding to the *Grm3* exon 4 region encoding the transmembrane domain, and the 5′ and 3′ arms were cloned and inserted into the targeting vector pflox, that carried a neo^r^ cassette and three *loxP* sites (Figure 1a). Homologous recombination at the *Grm3* locus in the mouse ES cells electroporated with the mGluR3 targeting vector was confirmed by Southern blot analysis (data not shown). The chimeric mice generated by the injection of correctly targeted ES cells into C57BL/6J blastocysts were crossed with C57BL/6 mice to obtain *Grm3*^targ/+^ offspring. Mice carrying the targeted allele were mated with *EIIa-Cre*^*Tg/0*^ mice to remove *Grm3* exon 4 and the neo^r^ gene. The resultant *Grm3*^null/+^ mice were intercrossed to obtain *Grm3*^null/null^ mice after backcross with C57BL/6 mice. The *Grm3*^null/null^ mice showed the 8.4 kb-*Bam*H I fragment on the 5′ probe and the 9.3 kb-*Sph* I fragment on the 3′ probe by Southern blotting (Figure 1b). Targeted disruption of the mGluR3 gene was also confirmed by RT-PCR of brain RNA from these mice (Figure 1c).

### Normal general characteristics of mGluR3 KO mice

A behavioral test battery was conducted as described previously [21,22]. There were no abnormalities evident in the physical aspects of mGluR3 KO mice, including reproductive capability. mGluR3 KO mice appeared to be normal and healthy, but mGluR3 KO mice weighed ~1.5 g less than WT littermates (Additional file 1: Figure S1a, Student’s t-test, genotype effect, t_37_ = 2.59, *p* = 0.0134). The body temperature, grip strength and wire hanging time of mGluR3 KO mice were normal (Additional file 1: Figure S1b-d).

### Hyper locomotor activity of mGluR3 KO mice

**Figure 2:**
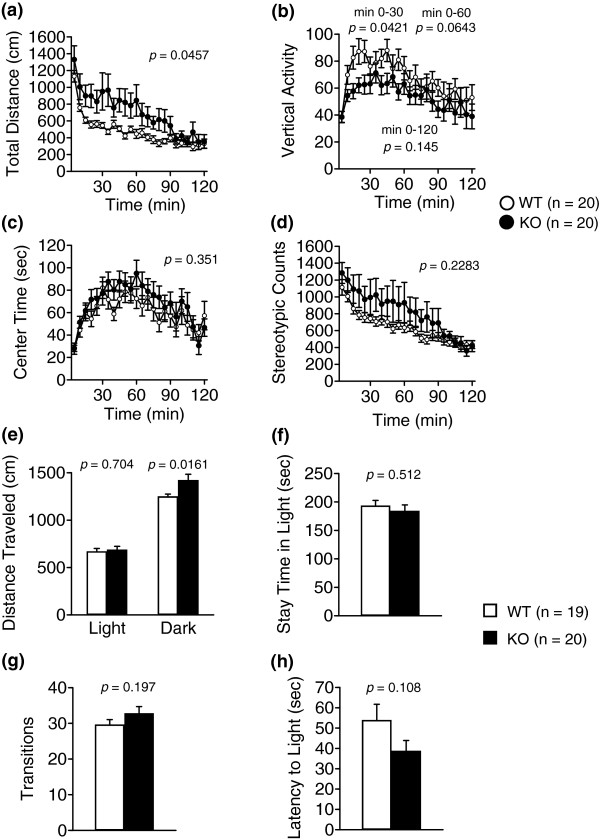
**Open field test and Light/dark transition test. (a-d)** Open field test: total distance traveled **(a)**, vertical activity **(b)**, time spent in the center of the compartment **(c)**, and stereotypic behavior **(d)** were recorded. The *p*-values indicate a genotype effect in the two-way repeated measures ANOVA (a-d, 0–120 minutes; b, 0–30 minutes, 0–60 minutes). Data are given as mean (±SEM). **(e-h)** Light/dark transition test: distance traveled in the light/dark compartments **(e)**, time spent in the light compartment **(f)**, number of light/dark transitions **(g)**, and latency to enter the light compartment **(h)** were recorded. The *p*-values indicate a genotype effect in the two-way repeated measures ANOVA **(a-d)** or Student’s t-test **(e-h)**. Data are given as mean (±SEM).

**Figure 3:**
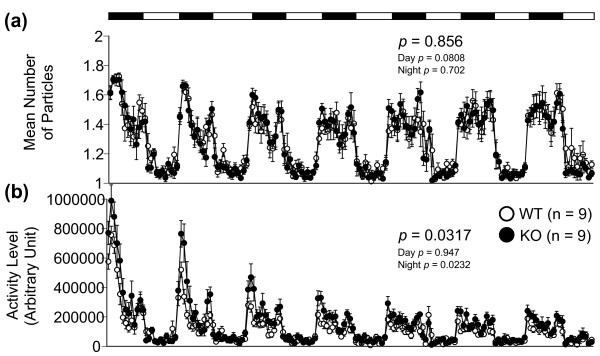
**Twenty-four hour home cage monitoring test.** Two animals of the same genotype were put in a cage, and their activity was monitored over ~ 7.5-day period. **(a)** Social interaction activity was represented as the number of particles in the image of the cage as viewed by automatic detection. When the animals were separated, two particles were seen, and when they were together, only one was observed. Social interaction in their home cage was not obviously different between genotypes. **(b)** The locomotor activity of KO mice was significantly greater than that of WT mice. Each dot indicates the mean per hour. The *p*-values indicate a genotype effect in the two-way repeated measures ANOVA. Data are given as mean (±SEM).

**Figure 4:**
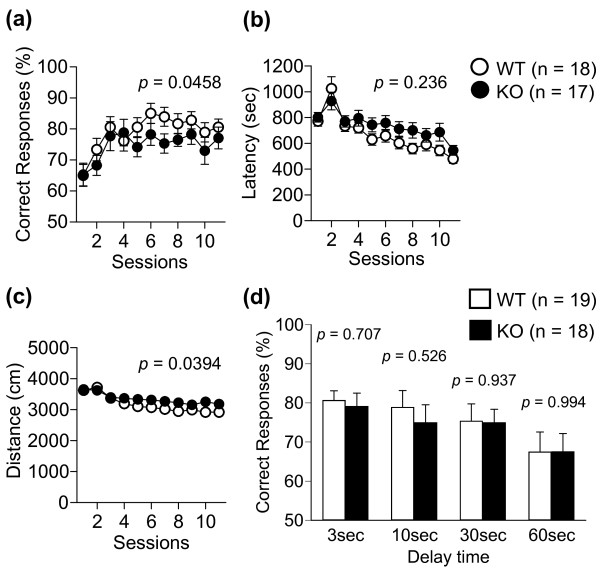
**T-maze forced alternation task test.** Correct response **(a)**, latency **(b)**, total distance **(c)**, and correct response at various latency times **(d)** were recorded. The *p*-values indicate a genotype effect in the two-way repeated measures ANOVA **(a-c)** or Student’s t-test **(d)**. Data are given as mean (±SEM).

Locomotor activity was examined in the open field (Figure 2a-d), light/dark transition (Figure 2e-h), 24-hour home cage monitoring (Figure 3), t-maze forced alternation task (Figure 4), elevated plus maze (Additional file 2: Figure S2), and social interaction tests (Additional file 3: Figure S3). mGluR3 KO mice traveled significantly longer distances in the open field test (Figure 2a, two-way repeated measures ANOVA, genotype effect, 0–120 minutes, F_1,38_ = 4.27, *p* = 0.0457), in the dark compartment in the light/dark transition test (Figure 2e, Student’s t-test, genotype effect, t_37_ = −2.52, *p* = 0.0161), and in the 24-hour home cage monitoring test (Figure 3b, two-way repeated measures ANOVA, genotype effect, F_1,16_ = 5.54, *p* = 0.0317) than those of WT mice. In contrast, decreased vertical activity was observed in mGluR3 KO mice in the open field test, especially at 0–30 minutes (Figure 2b, two-way repeated measures ANOVA, genotype effect, 0–30 minutes, F_1,38_ = 4.42, *p* = 0.0421). A significant difference was not observed in perseverative motor behavior in the stereotypic behavior of the open field test (Figure 2d). There was no significant difference in locomotor activity in the elevated plus maze (Additional file 2: Figure S2c), social interaction tests (Additional file 3: Figure S3e) and gait analysis (Additional file 4: Figure S4). T-maze forced alternation task test showed that distance traveled was significantly increased in KO mice (Figure 4c, two-way repeated measures ANOVA, genotype effect, F_1,33_ = 4.60, *p* = 0.0394), whereas the latency time was not different between the two genotypes (Figure 4b). Anxiety-like behaviors were examined in the light/dark transition, elevated plus maze and time spent in the center time of open field tests. There were no significant differences in these tests except for the distance traveled in the dark compartment in the light/dark transition test (Figure 2c, 2e-h), which indicated no difference in anxiety-like behaviors between the two genotypes.

### No impairment in social behavior of mGluR3 KO mice

Social behavior was examined in the 24-hour home cage monitoring test and in the social interaction test in a novel environment. There was no significant difference in the mean number of particles in the 24-hour home cage monitoring for 1 week (Figure 3a), and in all parameters of the social interaction test in a novel environment (Additional file 3: Figure S3).

### No impairment in prepulse inhibition of mGluR3 KO mice

The prepulse inhibition (PPI) test has been widely used to measure deficits in information-processing abilities or sensorimotor gating in schizophrenic patients [23], and can be employed in both human and animal experiments [24]. The PPI is defined as the degree (%) to which the acoustic startle response is reduced when the startle-eliciting stimulus is preceded by a brief, low-intensity, non-eliciting stimulus. There was no significant difference in the PPI test between the two genotypes (Additional file 5: Figure S5).

### Impaired reference and working memories of mGluR3 KO mice

**Figure 5:**
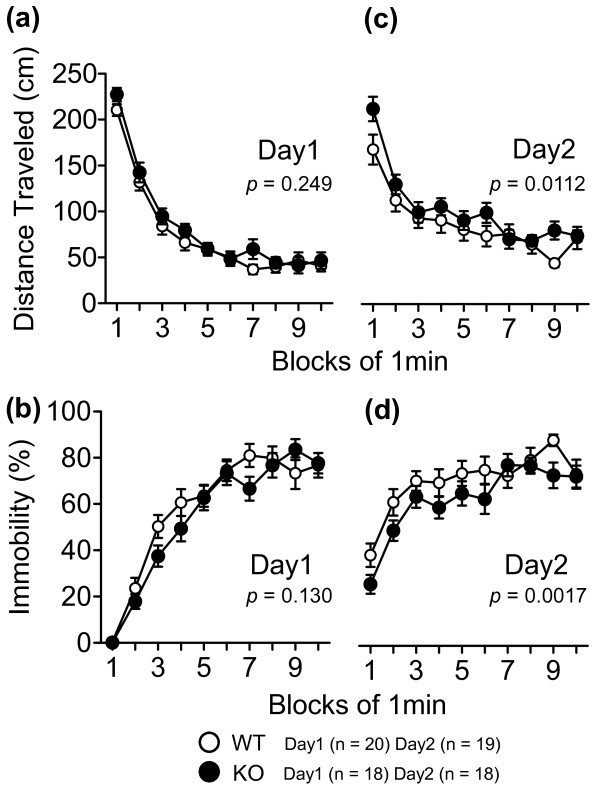
**Porsolt forced swim test.** Distance traveled **(a, c)** and immobility **(b, d)** were recorded in the Porsolt forced swim test for 2 trials. The *p-*values indicate a genotype effect in the two-way repeated measures ANOVA. Data are given as mean (±SEM).

In the Porsolt forced swim test, there was no significant difference between the two genotypes in the first trial (Figure 5a and b), indicating that mGluR3 KO mice did not show the depression-related behavior. However, mGluR3 KO mice traveled a greater distance (Figure 5c, two-way repeated measures ANOVA, genotype effect, F_1,35_ = 7.18, *p* = 0.0112) and showed lower immobility in the second trial, (Figure 5d, two-way repeated measures ANOVA, genotype effect, F_1,35_ = 11.6, *p* = 0.0017), suggesting that mGluR3 KO mice remembered the previous event less than WT mice. We analyzed the ratio of total distance traveled in each genotype (Day1 vs. Day2, respectively, Mann–Whitney *U*-test) and found that the ratio of total distance traveled was significantly increased in mGluR3 KO mice (*p* = 0.0449). These findings suggest that mGluR3 KO mice may have impaired reference memory for stressful events. Alternatively there is a possibility that depression-related behavior was simply decreased in mGluR3 KO mice on Day2. In the T-maze forced alternation task for working memory, mGluR3 KO mice had a significantly lower correct response than WT mice (Figure 4a, two-way repeated measures ANOVA for session 1–11, genotype effect, F_1,33_ = 4.31, *p* = 0.0458). To increase the difficulty of the task, a delay period (3, 10, 30, 60 seconds) was applied. Under these conditions, there was no significant difference in the percentage of the correct response between KO and WT mice (Figure 4d). These results suggested that working memory was slightly impaired in mGluR3 KO mice. However, there is also a possibility of impairment in reinforcement learning or procedural learning in the KO mice. There was no significant difference in spatial memory between KO and WT mice in the Barnes maze test (Additional file 6: Figure S6).

### Affected fear memory in mGluR3 KO mice

**Figure 6:**
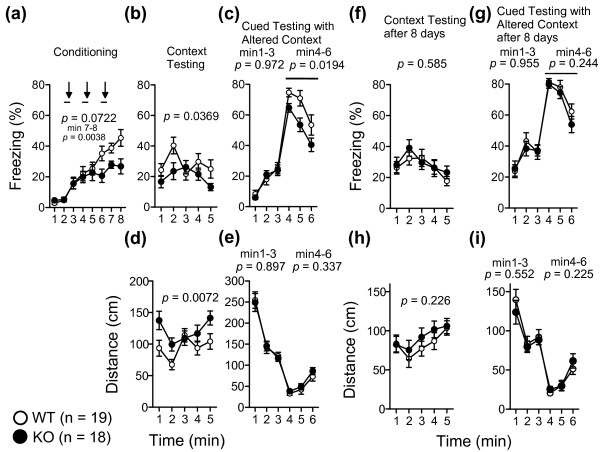
**Cued and contextual fear conditioning test.** Freezing rates during the conditioning phase **(a)**, contextual test performed 24-hours after conditioning **(b)** and the cued test with altered context **(c)** were recorded. Distance traveled in the contextual test **(d)** and cued test **(e)** 24-hours after conditioning were shown. The arrows and bars indicate the foot shock and the duration of the tone, respectively. Freezing during the contextual test conducted 8 days after conditioning **(f)** and the cued test with altered context 8 days after conditioning **(g)** were recorded. Distance in the contextual test **(h)** and cued test **(i)** 8 days after conditioning were shown. The *p*-values indicate a genotype effect in the two-way repeated measures ANOVA. Data are given as mean (±SEM).

The cognitive functions of KO and WT mice were examined in a cued and contextual fear conditioning test. Twenty-four hours after the conditioning session (Figure 6a), mGluR3 KO mice showed decreased levels of freezing during context testing (Figure 6b, two-way repeated measures ANOVA, genotype effect, F_1,35_ = 4.71, *p* = 0.0369; Figure 6d, two-way repeated measures ANOVA, genotype effect, F_1,35_ = 8.13, *p* = 0.0072) and cued testing with altered context testing at 4–6 minutes (Figure 6c, two-way repeated measures ANOVA, genotype effect, with tone, F_1,35_ = 6.00, *p* = 0.0194), although the distance was unaltered in mGluR3 KO mice in cued testing with altered context (Figure 6e). mGluR3 KO mice responded differently to the conditioning condition (Figure 6a, 7–8 min, *p* = 0.0038), suggesting that freezing level is simply decreasing in mGluR3 KO mice during sessions. There were no significant differences between KO and WT mice in the context and cued test with altered context 8 days after conditioning (Figure 6f-i). There was also no significant difference in fear memory between KO and WT mice in the passive avoidance test (Additional file 7: Figure S7). In the hot plate test, there was no significant difference between the two genotypes in the latency time (Additional file 8: Figure S8). These results suggested that contextual memory of stressful event, but not sensitivity to the stress may be affected in mGluR3 KO mice.

### Normal LTP of hippocampus of mGluR3 KO mice

**Figure 7:**
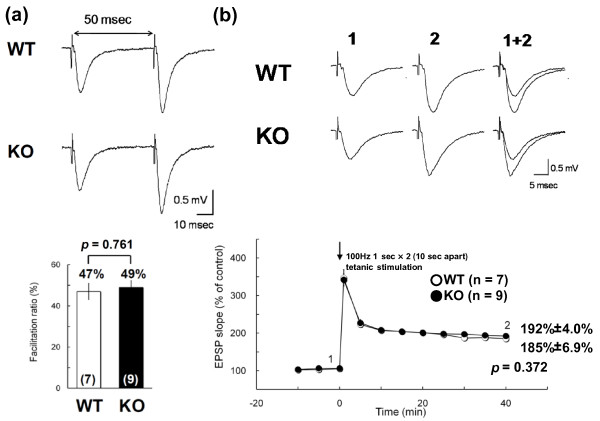
**Paired pulse facilitation and long-term potentiation. (a)** Facilitation ratios evaluated at 50-millisecond (msec) intervals; scale: 0.5 mV, 10 msec. The facilitation ratio values were computed as the ratio of the second stimulus-evoked fEPSP peak divided by the first stimulus-evoked fEPSP peak. The facilitation ratios of WT (white bar) and KO (black bar) hippocampal slices (WT: n = 7; KO: n = 9) were calculated from the averaged facilitation ratio values. **(b)** Average traces recorded at times 1 and 2; scale: 0.5 mV, 5 msec. The arrow indicates the time when the tetanic stimulation {100 Hz 1 sec × 2 (10 sec apart)} was applied. Regarding LTP, the normalized EPSP slope for WT mice 40 minutes after the tetanus was 185% ± 6.9% of the mean slope before stimulation, and that for KO mice was 192% ± 4.0%. There was no significant difference in the magnitude of LTP in WT and KO hippocampus slices (WT: n = 7; KO: n = 9) (*p* = 0.372). Data were given as mean (±SEM) and were analyzed with Student’s t-test.

We investigated short- and long-term synaptic plasticity by assessing paired-pulse facilitation (PPF) and long-term potentiation (LTP), respectively, in the CA1 region of the hippocampi of mice. The normalized facilitation ratio for WT mice (n = 7) was 47%, and that for KO mice (n = 9) was 49%, indicating that there were no differences in PPF between WT and KO mice (Figure 7a). These results showed that synaptic transmission was intact in mGluR3 KO mice. We, therefore, measured LTP in mGluR3 KO mice. Tetanic stimulation (100 Hz for 1 second delivered twice at 0.1 Hz) induced a long lasting increase in the synaptic strength of control slices (Figure 7a). The normalized EPSP slope for WT mice 40 minutes after the tetanus was 185% ± 6.9% of the average slope before stimulation (n = 7), and that for KO mice was 192% ± 4.0% (*p* = 0.372) (n = 9), indicating that there were no significant differences in LTP between the two genotypes (Figure 7b).

### Increased dopamine concentration in nucleus accumbens of mGluR3 KO mice

**Figure 8:**
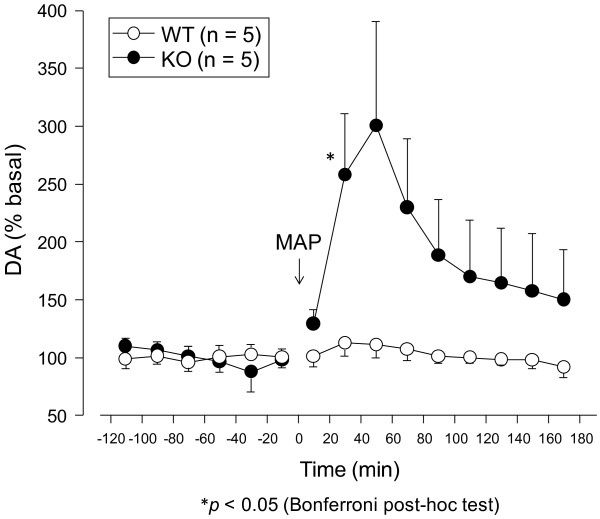
**In vivo microdialysis analysis of mGluR3 KO mice.** Methamphetamine (MAP) induced the release of dopamine in the nucleus accumbens of mGluR3 KO mice. The extracellular dopamine levels of WT and KO mice were determined by *in vivo* microdialysis and HPLC. After the collection of basal fractions, MAP (2 mg/kg, i.p) was administrated at time 0. Data are given as mean (±SEM) for the indicated number of mice. Repeated-measures analysis of variance revealed a genotype effect of F_1,88_ = 61.9 (*p <* 0.001), a time effect of F_8,82_ = 3.74 (*p <* 0.01), and a genotype × time effect of F_8,82_ = 2.49 (*p =* 0.02). *p < 0.05 (Bonferroni post-hoc test).

mGluR3 KO mice showed hyperactivity in comprehensive behavioral analyses. To investigate the molecular basis for the hyperactivity of mGluR3 KO mice, we conducted pharmaco-physiological analyses using the *in vivo* microdialysis procedure of mice. We measured the level of dopamine release induced by the intraperitoneal administration of MAP (2 mg/kg) in the nucleus accumbens. Sampling was performed during three hours following its administration. The MAP-induced increase in dopamine concentrations was significantly greater in the nucleus accumbens of KO mice than in WT mice (Figure 8). The low level MAP-induced increase of dopamine concentrations in the nuclear accumbens of WT mice could be due to difference in genetic background from the authentic WT mice because the WT mice prepared in this experiment were products of heterozygotes for the mGluR3 KO allele.

## Discussion

Certain behavioral abnormalities in KO mice of the relevant genes are similar to those observed in patients with psychiatric disorders [25,26]. mGluR3 KO mice showed hyperactivity and impaired working memory, which have been proposed as schizophrenia endophenotypes [27]. However, mGluR3 KO mice did not manifest other schizophrenia phenotypes such as PPI impairment and attenuated social behavior. Glutamatergic dysfunction due to the loss of mGluR3 is not sufficient to cause behavioral abnormalities related to the clinical symptoms of schizophrenia, which may require additional genetic factors and/or environmental factors to establish schizophrenia phenotypes because of the multifactorial background of the pathogenesis of schizophrenia. In fact, according to polygenic analysis of the results of genome-wide association studies, there may be thousands of common alleles with very small individual effects being involved in the risk of schizophrenia [28].

Group II mGluRs (mGluR2 and mGluR3) have key roles in synaptic plasticity, such as the modulation of LTP [6]. The results of the Porsolt forced swim test and cued and contextual fear conditioning test suggested impaired reference and contextual memories of stressful events in mGluR3 KO mice. To investigate the possible involvement of *Grm3* in reference and contextual memories through LTP, we performed electrophysiological analysis in the hippocampi of mice aged 12–14 weeks old. This age is almost the same age at which the Porsolt forced swim test was conducted. No significant difference in the magnitude of LTP was observed between the two genotypes. These results suggested that *Grm3* is not essential for LTP in the hippocampus, although hippocampal mossy fiber LTD was impaired in mGluR2 KO mice [29]. An inhibitory role of mGluR3 in LTP was observed in the rat dentate gyrus, both *in vitro*[7] and *in vivo*[8]. The mGluR3 specific agonist, NAAG (N-acetylaspartylglutamate) impaired the expression of LTP in the hippocampus, whereas its antagonist, β-NAAG had no effect [8], which is consistent with our results. A possibility of a compensatory effect of mGluR2 on LTP could not be excluded in mGluR3 KO mice. Ikegami *et al*. [30] reported that striatal dopamine D1 receptor is essential for contextual fear conditioning. The impaired fear conditioning in the mGluR3 KO mice could be due to dopaminergic dysfunction caused by a loss of mGluR3 in the hippocampal circuit.

Pharmaco-physiological analysis revealed that MAP-induced dopamine release was increased in mGluR3 KO mice. An increase in the extracellular levels of dopamine after cocaine administration was also observed in mGluR2 KO mice [31]. These observations suggest that group II mGluRs modulate the extracellular levels of dopamine underlying locomotor activity. This possibility has also been supported by *in vitro* and *in vivo* experimental evidence in which group II mGluRs negatively modulated dopamine release in the limbic and cortical regions [5]. Seeman *et al.*[32] revealed that D2 receptor was 17-fold more sensitive in mGluR3 knockout striate homogenates compared with control. There is also a possibility that D2 receptor hypersensitivity may cause hyper locomotor activity of mGluR3 mice in this study. The glutamate-dopamine interaction modulates the release of dopamine in the nucleus accumbens through the pyramidal neuron-GABA interneuron network of the prefrontal cortex and corresponding outputs to the nucleus accumbens [33], suggesting that a disturbance in the glutamate-dopamine interaction may be involved in the pathophysiology of schizophrenia-like behavior in mGluR3 KO mice. Further evaluations of the molecular and physiological properties of mGluR3 KO mice could provide new insights into the pathophysiology of schizophrenia and also the roles of mGluR3 in the higher brain function.

## Conclusions

mGluR3 KO mice showed hyperactivity and impaired working memories, both known as schizophrenia endophenotypes. MAP-induced dopamine release in the nucleus accumbens was increased in mGluR3 KO mice. *Grm3* may affect the extracellular level of dopamine underlying hyperactivity. These results demonstrate that a disturbance in the glutamate-dopamine interaction may be involved in the pathophysiology of schizophrenia-like behavior, such as hyperactivity in mGluR3 KO mice.

## Methods

### Generation of mGluR3 knockout mice

We disrupted exon 4 encoding the first six of seven transmembrane domains of mGluR3 of 129/Sv mice, although Corti *et al.*[34] reported a generation of mGluR3 mice by targeted disruption of exon 2. Genomic clones containing exon 4, which encodes the first six of seven transmembrane domains of mGluR3, were isolated from the 129/Sv mouse genomic library (Stratagene). To construct an mGluR3 targeting vector, the 5′ arm (4.4 kb), exon 4 region (2.5 kb), and 3′ arm (3.7 kb) fragments were isolated from the genomic clones and were inserted into the pflox vector. J1 embryonic stem (ES) cells derived from 129/SvJ strain mice were electroporated with a linearized mGluR3 targeting vector and cultured in medium containing 200 μg/ml G418. G418-resistant ES cells were genotyped by Southern blot analysis. Twelve recombinant ES cell clones were obtained from a total of 144 G418-resistant ES cell clones. The correctly targeted ES cell clones were injected into C57BL/6J (B6) blastocysts and subsequent chimeric mice were crossed with C57BL/6 mice to obtain *Grm3*^targ/+^ offspring. Female *Grm3*^targ/+^ mice were crossed with male transgenic mice expressing *Cre* recombinase under the control of the adenovirus *EIIa* promoter (*EIIa-Cre*) to obtain heterozygous mice carrying a null allele by the deletion of *Grm3* exon 4 and neo^r^ (neomycin-resistant) gene (*Grm3*^null/+^ mice). The *Grm3*^null/+^ offspring were further backcrossed with C57BL/6J mice for at least seven generations before behavioral, pharmacological and electrophysiological analyses. Finally, *Grm3*^null/+^ mice were intercrossed to generate homozygous knockout (KO, *Grm3*^null/null^) mice.

### Genotyping and sex determination

Genomic DNA was isolated from tail biopsies, and was used as a template for genotyping polymerase chain reaction (PCR). The primers used for the *Grm3* wild-type allele were: WT-F, 5′-GGG GGA AAT TTC ATC ATT CC-3′; and WT-KO-R, 5′-TGC TGT GCT TTC CTT TTG AG-3′; and for the null allele were: KO-F, 5′-TCT AGC CAA GAA CAC CAC CA-3′; and WT-KO-R. These primers amplified 411-bp and 329-bp fragments from the *Grm3* wild-type and null alleles, respectively. The sex determination of postnatal day 5 (P5) mice was also conducted by PCR analysis of tail genomic DNAs. The combination of a pair of primers was used to detect male mice: a pair of 5′-CCA TGT CAA GCG CCC CAT GA-3′ as the forward primer and 5′-GTA AGG CTT TTC CAC CTG CA-3′ as the reverse primer.

### Southern blot analysis

Mouse genomic DNA was extracted from G418-resistant ES cells, and was digested with restriction enzymes *Bam*H I and *Sph* I, fractionated by electrophoresis through 0.7% agarose gels, transferred onto Hybond-XL (GE Healthcare), and hybridized with the 5′ and 3′ probes, respectively. The 5′ probe detected 11.3 kb and 8.4 kb fragments corresponding to the wild-type and targeted alleles, respectively. The 3′ probe detected 11.6 kb and 7.8 kb fragments corresponding to the wild-type and recombinant alleles, respectively. The 5′ and 3′ probes used in Southern blot analysis corresponding to nucleotides 6519727–6520251 and 6504151–6504632 of NT_039299, respectively, were amplified from mouse genomic DNA by PCR. The nucleotide sequences of the primers for probes were as follows: 5′-TCG GGA TTC TTT CAT GCT GTT-3′ and 5′-AAT CAC CAG AGG GTC CTT CA -3′ were for the 5′ probe, and 5′-GCT TGC CTA TCC CCA AAA GT-3′ and 5′-CGG TGT TTC AGT CAC TGG TC -3′ were for the 3′ probe. The genomic DNA extracted from the mouse brain was also genotyped by Southern blotting to distinguish mice carrying the KO allele. The KO allele was detected as 8.4 kb *Bam*H I and 9.3 kb *Sph* I fragments by the 5′ and 3′ probes, respectively.

### Extraction of RNA and Reverse transcription-PCR (RT-PCR)

Prefrontal cortex tissues were collected from WT and mGluR3 KO mice. The mice were anesthetized with sevoflurane and decapitated. Brains were quickly removed under semi-sterile conditions. The brain tissues were retrieved from RNA*later* solution with sterile forceps, excess RNA*later* solution was quickly blotted away with an absorbent lab wipe, and the tissues were then submerged in ISOGEN (Nippongene). Tissues were homogenized promptly after placing them in ISOGEN on ice using a glass homogenizer potter. RNA was subsequently washed with 75% ethanol and suspended in diethylpyrocarbonate-treated water. Total RNA of 1 μg was reverse transcribed in a 20 μl reaction using the High Capacity RNA-to-cDNA Kit (ABI). The primer sequences for RT-PCR of *Grm3* were 5′-GCC ATT GAC AGC AGC AAC TA-3′ (exon 3) for the forward primer, and 5′-GCT TTG ACC AAG GGT GTG TT-3′ (exon 4) for the reverse primer.

### Comprehensive behavioral analyses

#### Animal and experiment design

Comprehensive behavioral experiments were done as previously described [21,22]. Prior to behavioral studies, mGluR3 KO males were obtained from the crossing between male and female N7 heterozygotes. The resulting heterozygotes were then intercrossed to produce homozygous WT and KO mice littermates. The behavioral tests were performed with the male mice of this generation, which were 10 weeks old at the start of the behavioral study (n = 20 for both groups). Mice were reorganized as soon as they were genotyped at the time of weaning, and were group-housed with four mice per cage, two WT and two KO littermates. Housing conditions included a 12-hour light/dark cycle, with lights on at 7:00 a.m. and access to food and water *ad libitum*. Behavioral testing was performed between 9:00 a.m. and 6:00 p.m. After the tests, all apparatus were cleaned with super hypochlorous water to prevent a bias based on olfactory cues with the apparatus. The order of each testing was as indicated in Table 1. Tests were conducted from least to more stressful tests, expect ones require long term recording periods like 24-hour homecage motoring. Animal experiments, including the production and maintenance protocols were approved by the Animal Research Committee of Kyushu University. All behavioral tests were approved by the Animal Research Committee of the National Institute for Physiological Sciences. Raw data from the behavioral tests, the date on which each experiment was performed, and the age of the mice at the time of the experiment are available in the Mouse Phenotype Database (http://www.mouse-phenotype.org/).

**Table 1:** Comprehensive behavioral test battery and the phenotypes of mGluR3 KO mice

**Tests**	**Phenotypes**	**Figures**	**Testing order**
General health			1
Whisker, coat, reflexes	-	N/A	
Somatic parameters			1
Body weight	↓	Additional file 1: Figure S1a	
Rectal temperature	-	Additional file 1: Figure S1b	
Grip strength	-	Additional file 1: Figure S1c	
Wire-hanging time	-	Additional file 1: Figure S1d	
Light/dark transition			
Anxiety	-	Figure 2e-h	2
Open field			
Exploratory locomotion	↑	Figure 2a-d	3
Elevated plus maze			
Anxiety	-	Additional file 2: Figure S2	4
Hot plate			
Pain sensitivity	-	Additional file 8: Figure S8	5
Social interaction test in a novel environment			
Sociability	-	Additional file 3: Figure S3	6
Rotarod			
Motor coordination	-	Not shown	7
Prepulse inhibition			
Sensorimotor gating	-	Additional file 5: Figure S5	8
Porsolt forced swim			
Immobility time (behavioral despair)	↓	Figure 5	9
Gait analysis			
Gait ataxia	-	Additional file 4: Figure S4	10
Barnes maze			
Spacial memory	-	Additional file 6: Figure S6	11
T-maze forced alternation task			
Working memory	↓	Figure 4	12
Cued and contextual fear conditioning			
Contextual fear memory	↓	Figure 6	13
Tail suspension			
Immobility time (behavioral despair)	-	Not shown	14
24-hour homecage monitoring			
Locomotor activity	↑	Figure 3	15
Passive avoidance			
Fear memory	-	Additional file 7: Figure S7	16

-, No significant differences between WT and KO; ↑, Higher in KO than in WT; ↓, Lower in KO than in WT. The testing order was indicated in the right column.

Rotarod test and tail suspension test were performed according to published methods [21].

### Open field test

Locomotor activity was measured using the open field test. The open field test was conducted as previously described [21]. Each subject was placed in the center of the open field apparatus (40 cm × 40 cm × 30 cm; Accuscan Instruments, Columbus, OH, USA). Total distance traveled (centimeters), vertical activity (rearing measured by counting the number of photobeam interruptions), time spent in the center, the beam-break counts for stereotypic behaviors, and the number of fecal boli were recorded. Data were collected for 120 minutes. Photo beams were positioned 1 cm inside apart from walls at the edge of the open field. The center area was defined as 20 cm × 20 cm area located at the center of the open field.

### Light/dark transition test

The apparatus used for the light/dark transition test consisted of a cage (21 cm × 42 cm × 25 cm) divided into two sections of equal size by a partition with a door (O’Hara & Co., Tokyo, Japan). One chamber was brightly illuminated (390 lx), whereas the other chamber was dark (2 lx). Mice were placed into the dark side, and allowed to move freely between the two chambers with the door open for 10 minutes. The total number of transitions, time spent in each compartment, first latency to the light side, and distance traveled were recorded automatically using ImageLD software. On-line material describing this method is available visually [35].

### Twenty-four hour home cage monitoring test

The 24-hour home cage monitoring test was conducted as previously described [36]. We used a system that automatically analyzed the locomotor activity of mice in the home cage. The system contained a home cage (29 cm × 18 cm × 12 cm) and a filter cage top, separated by a 13-cm-high metal stand containing an infrared video camera, which was attached to the top of the stand (O’Hara & Co., Tokyo, Japan). Two mice of the same genotype that had been housed separately were placed together in a home cage. Their locomotor activity and social behavior were monitored for 1 week. Outputs from the video cameras were fed into a computer and images from each cage were captured at a rate of one frame per second. Distance traveled was measured automatically using ImageHA software. Social interaction was measured by counting the number of particles detected in each frame: two particles indicted that the mice were not in contact with each other, and one particle (i.e., the tracking software could not distinguish two separate bodies) indicated contact between the two mice.

### T-maze forced alternation task test

The forced alternation task test was conducted using an automatic T-maze (O’Hara & Co., Tokyo, Japan) as described previously [37]. It was constructed of white plastic runways with 25 cm high walls. The maze was partitioned off into six areas by sliding doors that could be opened downward. The stem of the T was composed of area S2 (13 cm × 24 cm) and the arms of the T were composed of areas A1 and A2 (11.5 cm × 20.5 cm). Areas P1 and P2 were the connecting passage ways from the arms (area A1 or A2) to the start compartment (area S1). The end of each arm was equipped with a pellet dispenser that could provide a food reward. The pellet sensors were able to automatically record pellet intake by the mice. One week before pre-training, mice were deprived of food until their body weight was reduced to 80–85% of the initial level. Mice were kept on a maintenance diet throughout the course of all the T-maze experiments. Before the first trial, mice were subjected to three 10-minute adaptation sessions, during which they were allowed to freely explore the T-maze with all doors open and both arms baited with food. On the day after the adaptation session, mice were subjected to a forced alternation protocol for 11 days (one session consisting of 10 trials per day; cutoff time, 50 minutes). Mice were given 10 pairs of training trials per day. On the first (sample) trial of each pair, the mouse was forced to choose one of the arms of the T (area A1 or A2), and received the reward at the end of the arm. Choosing the incorrect arm resulted in no reward and confinement to the arm for 10 seconds. After the mouse consumed the pellet or the mouse stayed >10 seconds without consuming the pellet, the door that separated the arm (area A1 or A2) and connecting passage way (area P1 or P2) was opened and the mouse could return to the starting compartment (area S1), via the connecting passage way. The mouse was then given a 3-second delay there and a free choice between both T arms and rewarded for choosing the other arm that was not chosen on the first trial of the pair. The location of the sample arm (left or right) was varied pseudo-randomly across trials using the Gellermann schedule so that mice received equal numbers of left and right presentations. A variety of fixed extramaze cues surrounded the apparatus. A delay (10, 30, or 60 seconds) was applied three trials after the sample trial.

### Porsolt forced swim test

The Porsolt forced swim test was performed as described previously [38]. The apparatus consisted of four Plexiglas cylinders (20 cm height × 10 cm diameter; O’Hara & Co., Tokyo, Japan). The cylinders were filled with water at 23°C, up to a height of 7.5 cm. Mice were placed into the cylinders, and their behavior was recorded over a 10-minute test period (days 1 and 2). Images were captured at one frame per second. For each pair of successive frames, the amount of area (pixels) that the mouse moved in was measured. When the amount of area was below a certain threshold, mouse behavior was judged as “immobile.” When the amount of area equaled or exceeded the threshold, the mouse was considered as “moving.” The optimal threshold used for judging was determined through adjustments it to the amount of immobility measured by human observation. Immobility lasting for less than 2 seconds was not included in the analysis. Retention tests were administered 24 hours after training. Data acquisition and analysis were performed automatically using ImageTS software.

### Cued and contextual fear conditioning test

The cued and contextual fear conditioning test was conducted as previously described [39]. On the training day, each mouse was placed into a conditioning chamber (10.5 cm × 10.5 cm × 10.5 cm; O’Hara & Co., Tokyo, Japan) and allowed to explore freely for 2 minutes. A tone (75 dB) was presented as the conditioned stimulus for 30 seconds followed by a 2-second mild foot shock (0.3 mA) as the unconditioned stimulus. One or two more toneshock pairs were given at 2-minute intervals and the animal was returned to its home cage 30 seconds after the last pair. Twenty-four hours after the conditioning session, the mice were placed back into the conditioning chamber for 5 minutes and their freezing behavior was measured in context. One hour after the context testing, the mice were placed into a different, white Plexiglas chamber for 3 minutes and then the tone was turned on for 3 minutes. Freezing behavior was measured eight days after the conditioning in the same manner as that 24 hours after the conditioning. Data acquisition, control of stimuli (i.e., tones and shocks), and data analysis were performed automatically, using ImageFZ software. Images were captured at one frame per second. For each pair of successive frames, the amount of area (pixels) by which the mouse moved was measured. When this area was below a certain threshold (i.e., 20 pixels), the behavior was judged as ‘freezing’. When the amount of area equaled or exceeded the threshold, the behavior was considered as ‘non-freezing’. The optimal threshold (amount of pixels) to judge freezing was determined by adjusting it to the amount of freezing measured by human observation. ‘Freezing’ that lasted less than the defined time threshold (i.e., 2 s) was not included in the analysis.

### Image analysis

The applications used for the behavioral studies (ImageLD, ImageHA, ImageTM, ImageTS, ImageFZ, ImageEP, ImageSI, and ImageBM) were based on the NIH Image program (developed at the U.S. National Institutes of Health and available at http://rsb.info.nih.gov/nih-image) and the ImageJ program (http://rsb.info.nih.gov/ij). They were modified for each test by authors and are available through O’Hara & Co. ImageLD [35], ImageEP [40], ImageFZ [37] and ImageTM are freely available at the following URL: http://www.mouse-phenotype.org/software.html.

### Electrophysiological analysis

Electrophysiological analyses were performed as previously reported [22,41,42] in 12-14-week-old mice. Mouse brains were removed after sevoflurane anesthesia and decapitation. Transverse hippocampus slices were cut with a vibrating microtome (VT 1000 S) in ice-cold NR (normal Ringer Subject: solution saturated with 95% O_2_/5% CO_2_), and were incubated in NR to allow them to recover for at least 1 hour at room temperature. All recordings were carried out in a submerged slice chamber perfused with NR 33°C ± 0.5°C. A recording electrode filled with 0.9% NaCl was used for recording. Synaptic responses were evoked at 0.1 Hz using a bipolar tungsten electrode. Paired pulse facilitation (PPF) was examined at 50-millisecond (msec) interstimulus intervals with three traces. PPF values were calculated as the ratio of the second stimulus-evoked field excitatory postsynaptic potential (fEPSP) peak divided by the first stimulus-evoked fEPSP peak. Frequency facilitation was calculated to obtain the average value of the facilitation ratio of three traces. Tetanic stimulation to evoke LTP consisted of two trains of 100 Hz stimulation lasting for 1 second at an inter-trial interval of 10 seconds. LTP of the fEPSP slope was expressed as a percentage of the mean before tetanic stimulation. To preclude bias, the experiments of the PPF and LTP measurements were blinded with respect to the genotype of the mice analyzed. All data were expressed as a mean ± SEM analyzed with Student’s t-test.

### Pharmaco-physiological analysis

Pharmaco-physiological analysis was conducted as previously described [43]. We performed *in vivo* microdialysis measurements of extracellular dopamine in the central nervous systems of 10-12-week-old mice. These mice were anesthetized with sodium pentobarbital, and a guide cannula (AG-4; EICOM, Kyoto, Japan) was implanted into the nucleus accumbens (+1.1 mm anteroposterior and +1.0 mm mediolateral relative to the bregma and −4.0 mm dorsoventral relative to the dura of the skull, according to the atlas of Franklin and Paxinos) and was secured to the skull with stainless steel screws and dental acrylic cement. The placement of probes were confirmed by the standard histological examination. One day after the surgery, a dialysis probe (AI-4-1, 1-mm membrane length; EICOM) was inserted through the guide cannula and was perfused at a flow rate of 1 μl/min with artificial cerebrospinal fluid (148 mM NaCl, 2.7 mM KCl, 1.2 mM CaCl_2_, 0.85 mM MgCl_2_). Sample collection was started after an equilibration period of 1 hour. Outflow fractions were collected every 20 minutes. After the collection of at least six baseline fractions, mice were treated with MAP (2 mg/kg, i.p.) and sampling was continued for an additional 300 minutes. The amount of dopamine in the dialysis fractions was measured by high-performance liquid chromatography (HPLC) on PP-ODS column (EICOM) that was maintained at 25°C and equipped with an electrochemical detection system (HTEC-500, EICOM) and PowerChrom (EICOM). The mobile phase comprised 0.1 M phosphate buffer (pH 6.0) containing 1% methanol, sodium decanesulfonic acid (500 mg/l), and EDTA (disodium salt, 50 mg/l) and was delivered at a flow rate of 0.5 ml/minute.

### Statistical analysis

Statistical analyses of comprehensive behavioral tests were conducted by using StatView (SAS Institute, Cary, NC). Data were analyzed by Student’s t-test or Mann–Whitney *U*-test, one-way Analysis of Variance (ANOVA) or two-way repeated measures ANOVA. Values in graphs are expressed as mean ± SEM. Electrophysiological analysis was performed using the Student’s t-test. Pharmaco-physiological analysis was conducted using the Bonferroni post-hoc test.

The detailed method for statistical analysis was described in the Figure legend.

The following methods of behavioral analyses, whose results were shown in Additional files 1, 2, 3, 4, 5, 6, 7 and 8.

### General health and neurological examination

To compare the physical characteristics of WT mice and KO mice littermates, we first conducted neurological screening as previously described [38]. The righting, whiskers touch, and ear twitch reflexes were evaluated, and a number of physical features including body weight and temperature, and the presence of whiskers or bald hair patches were recorded. Neuromuscular strength was examined by the grip strength and wire-hanging tests. The grip strength meter (O’Hara & Co., Tokyo, Japan) was used to assess forelimb grip strength. Mice were lifted and held by their tail so that their forepaws could grasp a wire grid. Mice were then gently pulled backward by the tail with their posture parallel to the surface of the table until they released the grid. The peak force applied by the mouse forelimbs was recorded in newtons (N). Each mouse was tested three times and the greatest value measured was used for statistical analysis. In the wire hanging test, mice were placed on a wire mesh, which was then inverted and waved gently, so that the subject gripped the wire. Latency to fall was recorded, with a 60-second cut-off time.

### Elevated plus maze test

The elevated plus-maze consisted of two open arms (25 cm × 5 cm) and two enclosed arms of the same size, with 15 cm high transparent walls. The arms and central square were made of white plastic plates, and were elevated to a height of 55 cm above the floor. In order to minimize the likelihood of animals falling from the apparatus, 3 mm high Plexiglas ledges were provided for the open arms. Arms of the same type were arranged at opposite sides to each other. Each mouse was placed in the central square of the maze (5 cm × 5 cm), facing one of the closed arms. Mouse behavior was recorded during a 10-minute test period as previously described [40]. The number of entries into, and the time spent on open and enclosed arms were recorded. For data analysis, we employed the following four measures: the percentage of entries into the open arms, the time stayed in the open arms (seconds), the number of total entries, and total distance traveled (centimeters). Data acquisition and analysis were performed automatically, using ImageEP software.

### Social interaction test in a novel environment

The social interaction test in a novel environment was conducted as previously described [44]. Two mice of identical genotypes, which were previously housed in different cages, were placed into a box together (40 cm × 40 cm × 30 cm) and allowed to explore it freely for 10 minutes. Social behavior was monitored by a CCD camera, which was connected to a Macintosh computer. Analysis was performed automatically using ImageSI software. The number of contacts, duration of contacts, and total distance traveled were measured.

### Gait analysis

The gait analysis was conducted as previously described [45]. Digital video images of the underside of mice were collected at 150 frames per second with a high-speed video camera from below the transparent belt of a motorized treadmill (DigiGait Imaging System, Mouse Specifics, Quincy, MA, USA). The compartment of the treadmill in which the mouse walked was ~25 cm in length and ~5 cm wide, and could be changed to accommodate different-sized animals. The software of DigiGait Imaging System identified the four paws of each mouse in each individual image of the underside of the mouse as it walked on the belt through its strides. Each image represented 6.67 msec; the paw area indicated the temporal placement of the paw relative to the treadmill belt. Color images were converted to their binary matrix equivalents, and the areas (in pixels) of the approaching or retreating paws relative to the belt and camera were calculated throughout each stride. Plotting the area of each digital paw print (paw contact area) imaged sequentially in time provided a dynamic gait signal, representing the temporal record of paw placement relative to the treadmill belt. Each gait signal for each limb comprised a stride duration (stride time), which included the stance duration when the paw of a limb was in contact with the walking surface, plus the swing duration when the paw of the same limb is not in contact with the walking surface. Stance duration was further subdivided into braking duration (increasing paw contact area over time) and propulsion duration (decreasing paw contact area over time). Stride frequency was calculated by counting the number of gait signals over time. Stride length was calculated from the equation: *speed = stride frequency × stride length*. To obtain stance widths and paw placement angles at full stance, ellipses were fitted to the paws, and the centers, vertices, and major axes of the ellipses were determined. Forelimb and hindlimb stance widths were calculated as the perpendicular distance between the major axes of the left and right paw images during peak stance. Gait data were collected and pooled from both the left and right forelimbs, and the left and right hindlimbs.

### Startle response/prepulse inhibition

The startle response/prepulse inhibition test was conducted as previously described [44]. A startle reflex measurement system was used (O’Hara & Co., Tokyo, Japan). A test session began by placing a mouse in a Plexiglas cylinder where it was left undisturbed for 10 minutes. The duration of white noise that was used as the startle stimulus was 40 msec for all trial types. The startle response was recorded for 140 msec (measuring the response every 1 msec) starting with the onset of the prepulse stimulus. The background noise level in each chamber was 70 dB. The peak startle amplitude recorded during the 140 msec sampling window was used as the dependent variable. A test session consisted of six trial types [i.e., two types for ‘startle-stimulus-only’ trials, and four types for prepulse inhibition (PPI) trials]. The intensity of startle stimulus was 110 or 120 dB. The prepulse sound was presented 100 msec before the startle stimulus, and its intensity was 74 or 78 dB. Four combinations of prepulse and startle stimuli were employed (74–110, 78–110, 74–120, and 78–120 dB). Six blocks of the six trial types were presented in pseudo-random order such that each trial type was presented once within a block. The average inter-trial interval was 15 seconds (range: 10–20 seconds).

### Barnes maze test

The Barnes maze test was performed as described previously [21]. The test was conducted on “dry land”, a white circular surface, 1.0 m in diameter, with 12 holes equally spaced around the perimeter (O’Hara & Co., Tokyo, Japan). The circular open field was elevated 75 cm from the floor, and evenly illuminated by overhead fluorescent white room lighting (1000 lux). A black Plexiglas escape box (17 cm × 13 cm × 7 cm) containing shredded paper was located under one of the holes. The hole above the escape box represented the target, analogous to the hidden platform in the Morris task. The location of the target was consistent for a given mouse, but was randomized across mice. The maze was rotated daily, with the spatial location of the target was unchanged with respect to the distal visual room cues, to prevent a bias based on olfactory or proximal cues within the maze. Eighteen trials were conducted. One day after the last training, a probe test was conducted without the escape box, to confirm that this spatial task was acquired based on navigation using distal environment room cues. One trial was conducted immediately after the probe test, and additional probe tests were conducted again 1 month later. Latency, the number of errors, distance traveled until they located the target hole, and the time spent around each hole were recorded by ImageBM software.

### Passive avoidance test

Passive avoidance test was conducted as described previously [21]. The apparatus was a trapezoidal box, consisting of one dark and one bright chamber connected by a guillotine door (O’Hara & Co., Tokyo, Japan). Each mouse was first placed into the lighted chamber and the guillotine door was opened. After mouse entered the dark chamber, a two seconds footshock at 0.3 mA was delivered to mouse. Mice that did not enter the dark chamber within 300 seconds were excluded from analysis. One day and two days later, animals were tested for retention by placing each animal into the lighted chamber and the latency of the mouse entering the dark chamber was recorded.

### Hot plate test

The hot plate test was conducted as previously described [21]. The hot plate test was used to evaluate nociception or sensitivity to a painful stimulus. Mice were placed on a hot plate at 55.0 ± 0.3°C (Columbus Instruments, Columbus, OH, USA), and latency to the first hind-paw response was recorded. The hind-paw response was either a foot shake or paw lick.

## Abbreviations

mGluR3: Metabotropic glutamate receptor 3; KO: Knockout; WT: Wild-type; mGluRs: Metabotropic glutamate receptors; ES: Embryonic stem; B6: C57BL/6J; EIIa-Cre: *Cre* recombinase under the control of the adenovirus *EIIa* promoter; neor: Neomycin-resistant; PCR: Polymerase chain reaction; RT-PCR: Reverse transcription-PCR; NR: Normal ringer; LTP: Long-term potentiation; PPF: Paired pulse facilitation; fEPSP: Field excitatory postsynaptic potential; MAP: Methamphetamine; PPI: Prepulse inhibition; NAAG: N-acetylaspartylglutamate; GABA: Gamma-aminobutyric acid.

## Competing interests

The authors declare that they have no competing interests.

## Authors’ contributions

RF and TN co-wrote the manuscript. RF carried out the electrophysiological analyses under the supervision of II. TN performed the comprehensive behavioral experiments under the supervision of SH, KT, and TM. AI, AS and MN participated in generation and characterization of mGluR3 KO mice. KK conducted the pharmaco-physiological analyses. YF designed the study. All authors read and approved the final manuscript.

## Supplementary Material

Additional file 1: Figure S1 Somatic parameters and general behavior. Body weight **(a)**, body temperature **(b)**, grip strength **(c)**, and latency to fall in the wire hang test **(d)** were recorded. The *p*-values indicate a genotype effect in Student’s t-test. Data are given as mean (±SEM). Click here for file

Additional file 2: Figure S2 Elevated plus maze test. The number of entries into the center crossing between the open and closed arms **(a)**, number of entries into the open arms **(b)**, distance traveled **(c)**, the total time spent in the open arms **(d)**, the total time spent in the close arms **(e)**, and the total time spent in the center **(f)** were recorded. The *p*-values indicate a genotype effect in the one-way ANOVA. Data are given as mean (±SEM). Click here for file

Additional file 3: Figure S3 Social interaction test in a novel environment. The total duration of contacts **(a)**, number of contacts **(b)**, total duration of active contacts **(c)**, mean duration of each contact **(d)**, and the total distance traveled **(e)** were recorded. The *p*-values indicate a genotype effect in the one-way ANOVA. Data are given as mean (±SEM). Click here for file

Additional file 4: Figure S4 Gait analysis. **(a-f)** Front paw and **(g-l)** hind paw. Stride duration of swing, brake, and propel **(a, g)**, stance duration of brake and propel **(b, h)**, stance width **(c, i)**, stride length **(d, j)**, step angle **(e, k)**, and paw angle **(f, l)** were recorded. The *p*-values indicate a genotype effect in the one-way ANOVA. Data are given as mean (±SEM). There were no significant differences excepting for stride length of front and hind paws (**d**, *p* = 0.0182; **j**, *p* = 0.0087), and paw angle of hind paw (**l**, *p* = 0.0119). It is possible that the weight difference is a cause in the difference of the stride length. Click here for file

Additional file 5: Figure S5 Startle response/prepulse inhibition. The acoustic startle response **(a)** and prepulse inhibition test **(b)** were recorded. The *p*-values indicate a genotype effect in the one-way ANOVA. Data are given as mean (±SEM). Click here for file

Additional file 6: Figure S6 Barnes maze test. **(a, b)** Training course: Latency to the target hole **(a)** and the number of errors to the target hole **(b)** were recorded. **(c, d)** Probe tests 24-hours after the last training: Time spent around each hole **(c)** and the ratio of time spent around the target and target + ±30 **(d)** were recorded. **(e, f)** Probe tests 1 month after the last training: time spent around each hole **(e)**, ratio of time spent around the target and target + ±30 **(f)** were recorded. The *p*-values indicate a genotype effect in the two-way repeated measures ANOVA **(a, b)**, one-way ANOVA **(c, e)** and Mann-Whitney *U*-test **(d, f)**. Data are given as mean (±SEM). Click here for file

Additional file 7: Figure S7 Passive avoidance test. **(a, b)** Latency to enter dark compartment after one day **(a)** and two days **(b)** were recorded. The *p*-values indicate a genotype effect in the one-way ANOVA. Data are given as mean (±SEM). Click here for file

Additional file 8: Figure S8 Hot plate test. Latency to the first hind-paw response was recorded. The *p*-values indicate a genotype effect in the one-way ANOVA. Data are given as mean (±SEM). Click here for file
