# Supplementary material for: Comprehensive behavioral study of mGluR3 knockout mice: implication in schizophrenia related endophenotypes
Source: Mol Brain. 2014 Apr 23;7:31. doi: 10.1186/1756-6606-7-31 (PMC4021612; doi:10.1186/1756-6606-7-31)
Supplement: Additional file 1: Figure S1 — Somatic parameters and general behavior. Body weight (a), body temperature (b), grip strength (c), and latency to fall in the wire hang test (d) were recorded. The p-values indicate a genotype effect in Student’s t-test. Data are given as mean (±SEM). [file 1756-6606-7-31-S1.pdf]

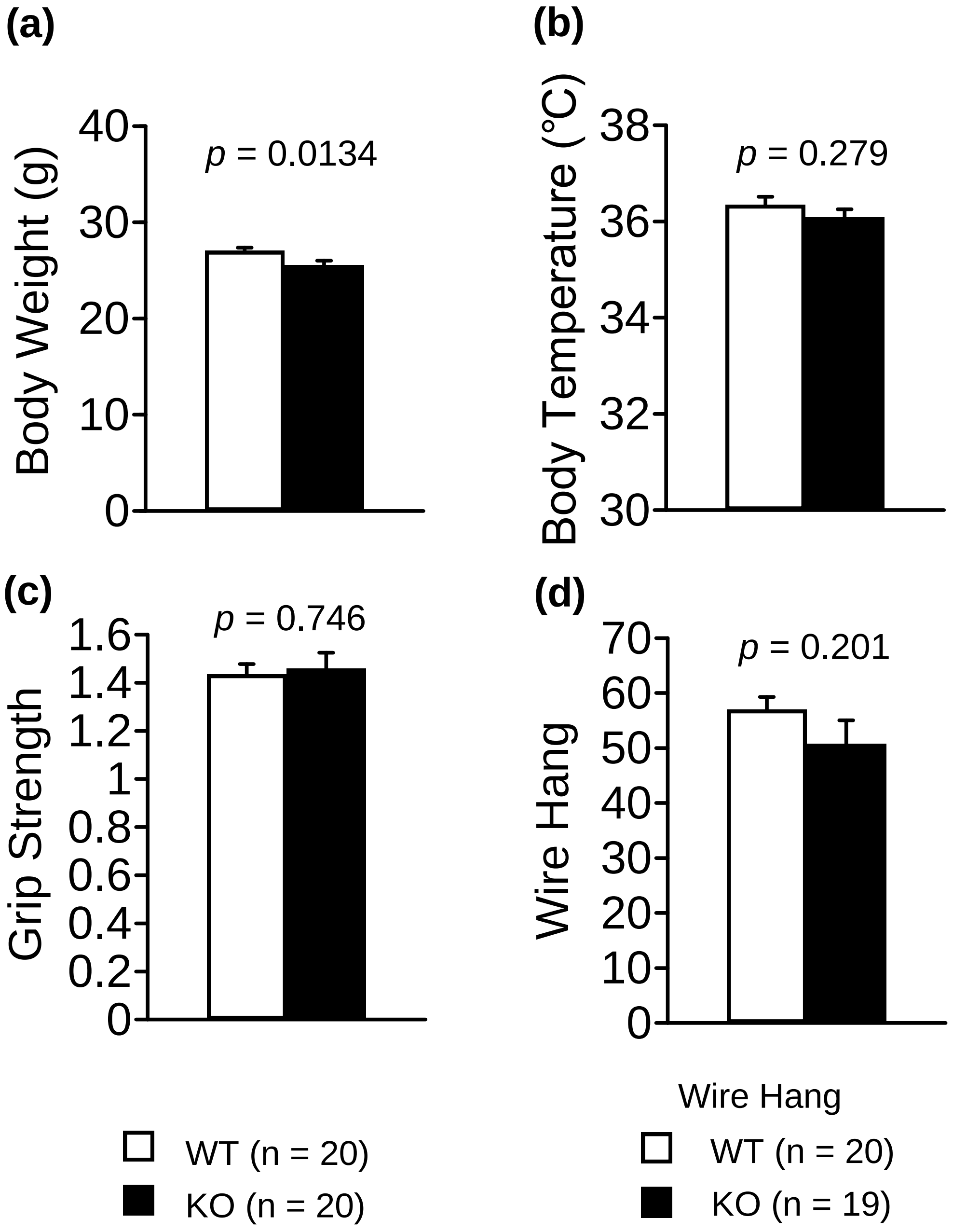

Supplemental Figure S1: Somatic parameters and general behavior. Body weight (a), body temperature (b), grip strength (c), and latency to fall in the wire hang test (d) were recorded. The *p*-values indicate a genotype effect in Student's t-test. Data are given as mean (±SEM).
