# Supplementary material for: Comprehensive behavioral study of mGluR3 knockout mice: implication in schizophrenia related endophenotypes
Source: Mol Brain. 2014 Apr 23;7:31. doi: 10.1186/1756-6606-7-31 (PMC4021612; doi:10.1186/1756-6606-7-31)
Supplement: Additional file 2: Figure S2 — Elevated plus maze test. The number of entries into the center crossing between the open and closed arms (a), number of entries into the open arms (b), distance traveled (c), the total time spent in the open arms (d), the total time spent in the close arms (e), and the total time spent in the center (f) were recorded. The p-values indicate a genotype effect in the one-way ANOVA. Data are given as mean (±SEM). [file 1756-6606-7-31-S2.pdf]

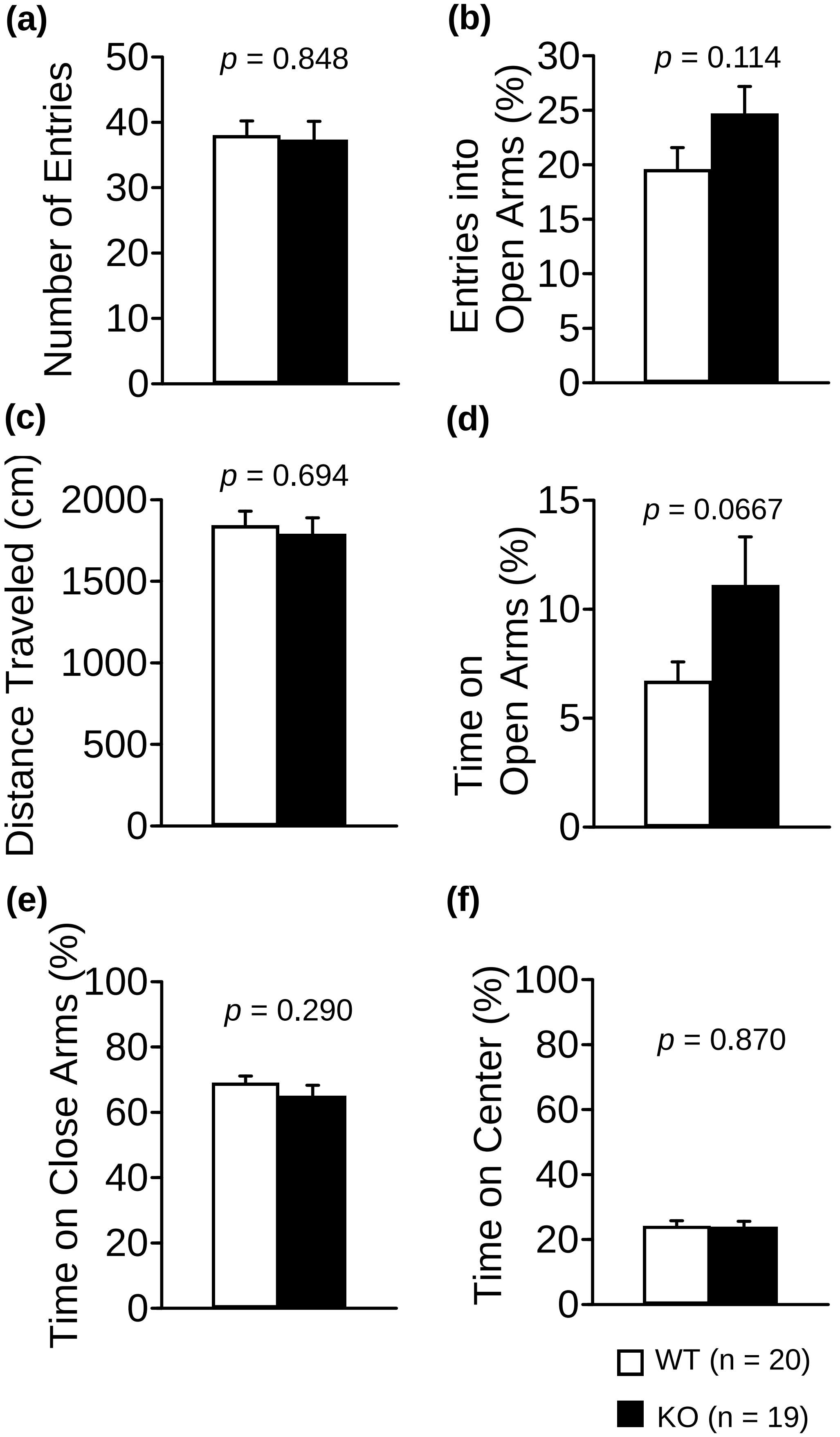

Supplemental Figure S2: Elevated plus maze test. The number of entries into the center crossing between the open and closed arms (a), number of entries into the open arms (b), distance traveled (c), the total time spent in the open arms (d), the total time spent in the close arms (e), and the total time spent in the center (f) were recorded. The *p*-values indicate a genotype effect in the one-way ANOVA. Data are given as mean (±SEM).
