# Supplementary material for: Comprehensive behavioral study of mGluR3 knockout mice: implication in schizophrenia related endophenotypes
Source: Mol Brain. 2014 Apr 23;7:31. doi: 10.1186/1756-6606-7-31 (PMC4021612; doi:10.1186/1756-6606-7-31)
Supplement: Additional file 3: Figure S3 — Social interaction test in a novel environment. The total duration of contacts (a), number of contacts (b), total duration of active contacts (c), mean duration of each contact (d), and the total distance traveled (e) were recorded. The p-values indicate a genotype effect in the one-way ANOVA. Data are given as mean (±SEM). [file 1756-6606-7-31-S3.pdf]

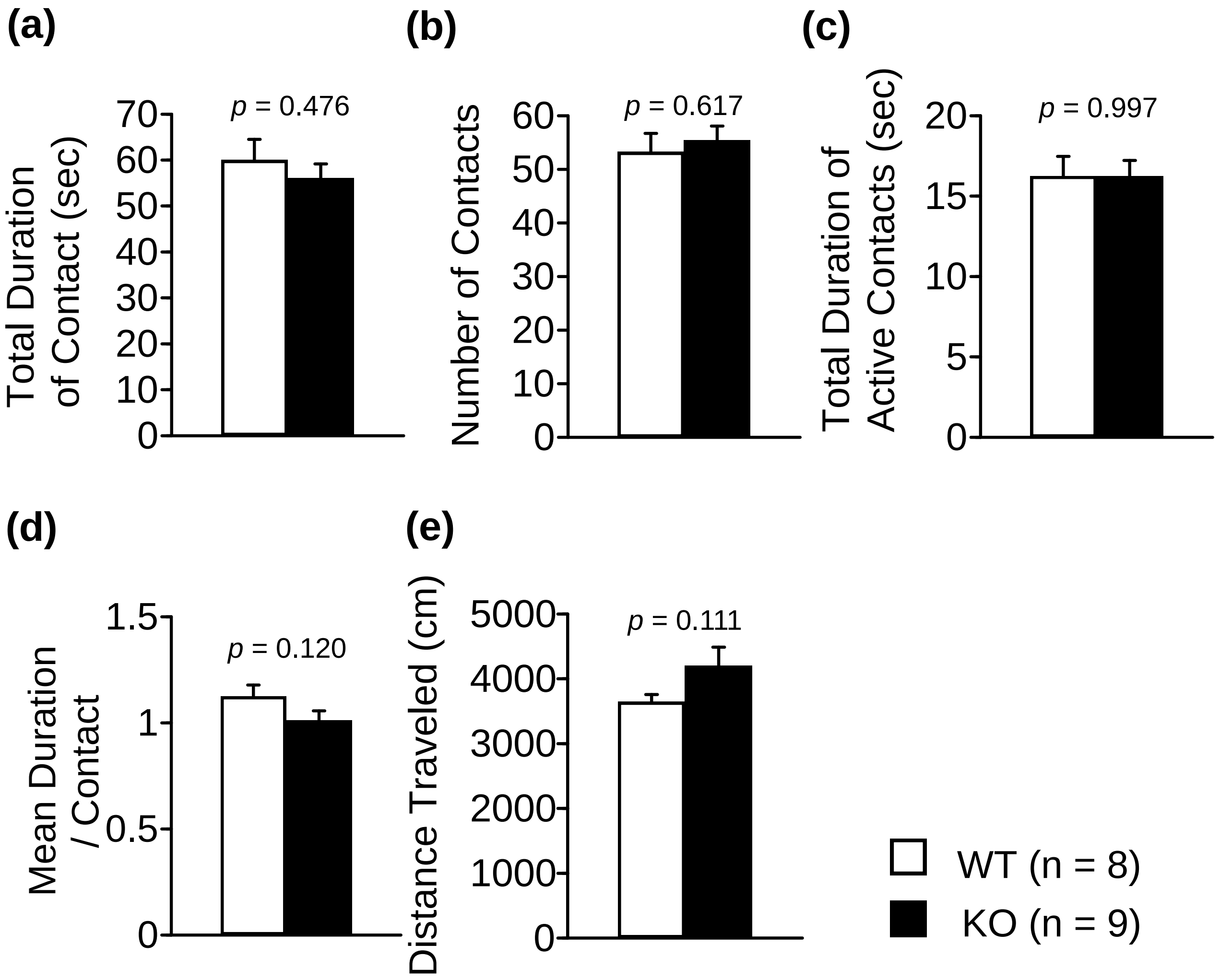

Supplemental Figure S3: Social interaction test in a novel environment. The total duration of contacts (a), number of contacts (b), total duration of active contacts (c), mean duration of each contact (d), and the total distance traveled (e) were recorded. The *p*-values indicate a genotype effect in the one-way ANOVA. Data are given as mean (±SEM).
