# Supplementary material for: Comprehensive behavioral study of mGluR3 knockout mice: implication in schizophrenia related endophenotypes
Source: Mol Brain. 2014 Apr 23;7:31. doi: 10.1186/1756-6606-7-31 (PMC4021612; doi:10.1186/1756-6606-7-31)
Supplement: Additional file 4: Figure S4 — Gait analysis. (a-f) Front paw and (g-l) hind paw. Stride duration of swing, brake, and propel (a, g), stance duration of brake and propel (b, h), stance width (c, i), stride length (d, j), step angle (e, k), and paw angle (f, l) were recorded. The p-values indicate a genotype effect in the one-way ANOVA. Data are given as mean (±SEM). There were no significant differences excepting for stride length of front and hind paws (d, p = 0.0182; j, p = 0.0087), and paw angle of hind paw (l, p = 0.0119). It is possible that the weight difference is a cause in the difference of the stride length. [file 1756-6606-7-31-S4.pdf]

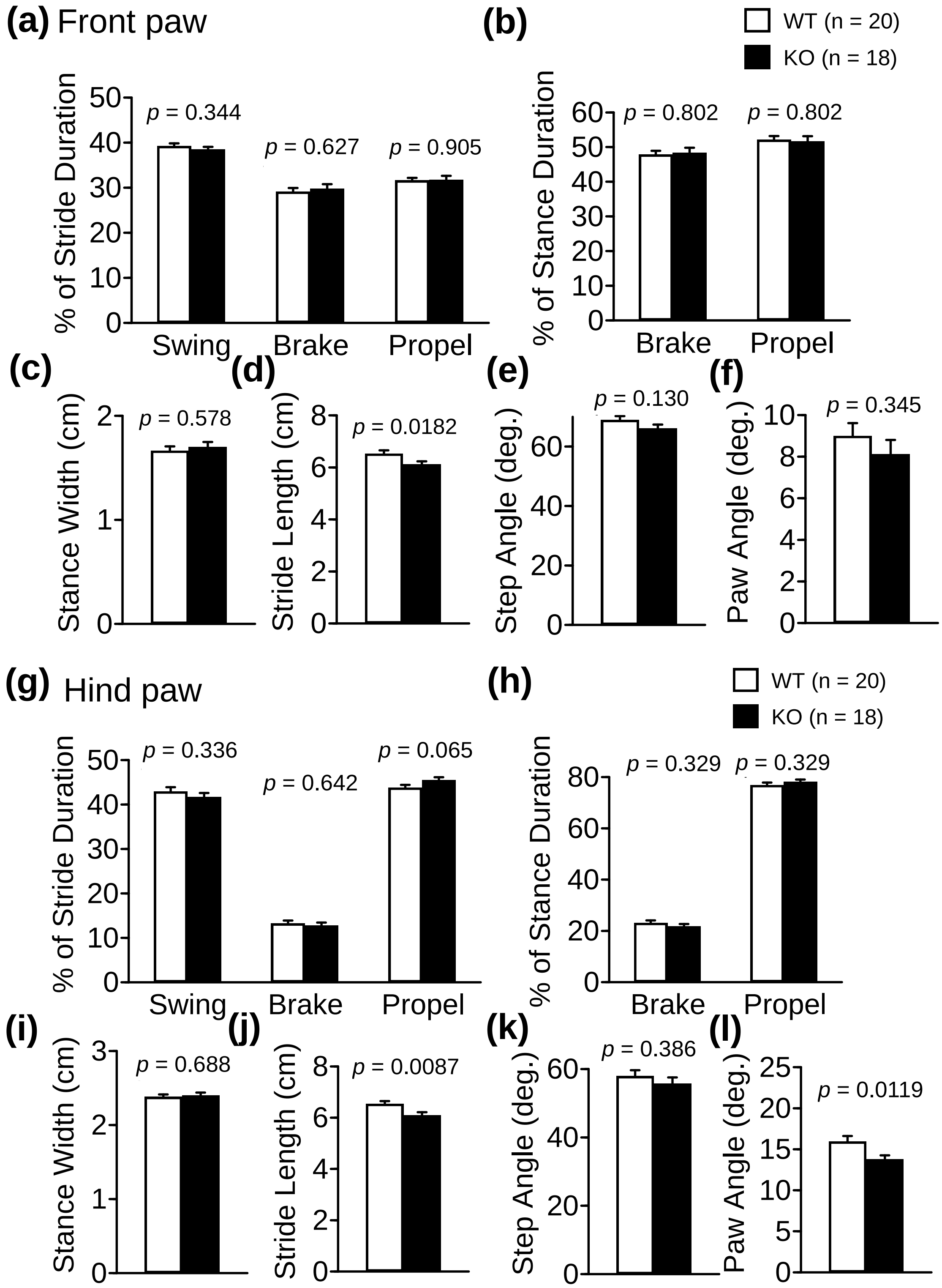

Supplemental Figure S4: Gait analysis. (a-f) Front paw and (g-l) hind paw. Stride duration of swing, brake, and propel (a, g), stance duration of brake and propel (b, h), stance width (c, i), stride length (d, j), step angle (e, k), and paw angle (f, l) were recorded. The *p*-values indicate a genotype effect in the one-way ANOVA. Data are given as mean (±SEM). There were no significant differences excepting for stride length of front and hind paw (d, p = 0.0182; j, p = 0.0087), and paw angle of hind paw (l, p = 0.0119). It is possible that the weight difference is a cause in the difference of the stride length.
