# Supplementary material for: Comprehensive behavioral study of mGluR3 knockout mice: implication in schizophrenia related endophenotypes
Source: Mol Brain. 2014 Apr 23;7:31. doi: 10.1186/1756-6606-7-31 (PMC4021612; doi:10.1186/1756-6606-7-31)
Supplement: Additional file 6: Figure S6 — Barnes maze test. (a, b) Training course: Latency to the target hole (a) and the number of errors to the target hole (b) were recorded. (c, d) Probe tests 24-hours after the last training: Time spent around each hole (c) and the ratio of time spent around the target and target + ±30 (d) were recorded. (e, f) Probe tests 1 month after the last training: time spent around each hole (e), ratio of time spent around the target and target + ±30 (f) were recorded. The p-values indicate a genotype effect in the two-way repeated measures ANOVA (a, b), one-way ANOVA (c, e) and Mann-Whitney U-test (d, f). Data are given as mean (±SEM). [file 1756-6606-7-31-S6.pdf]

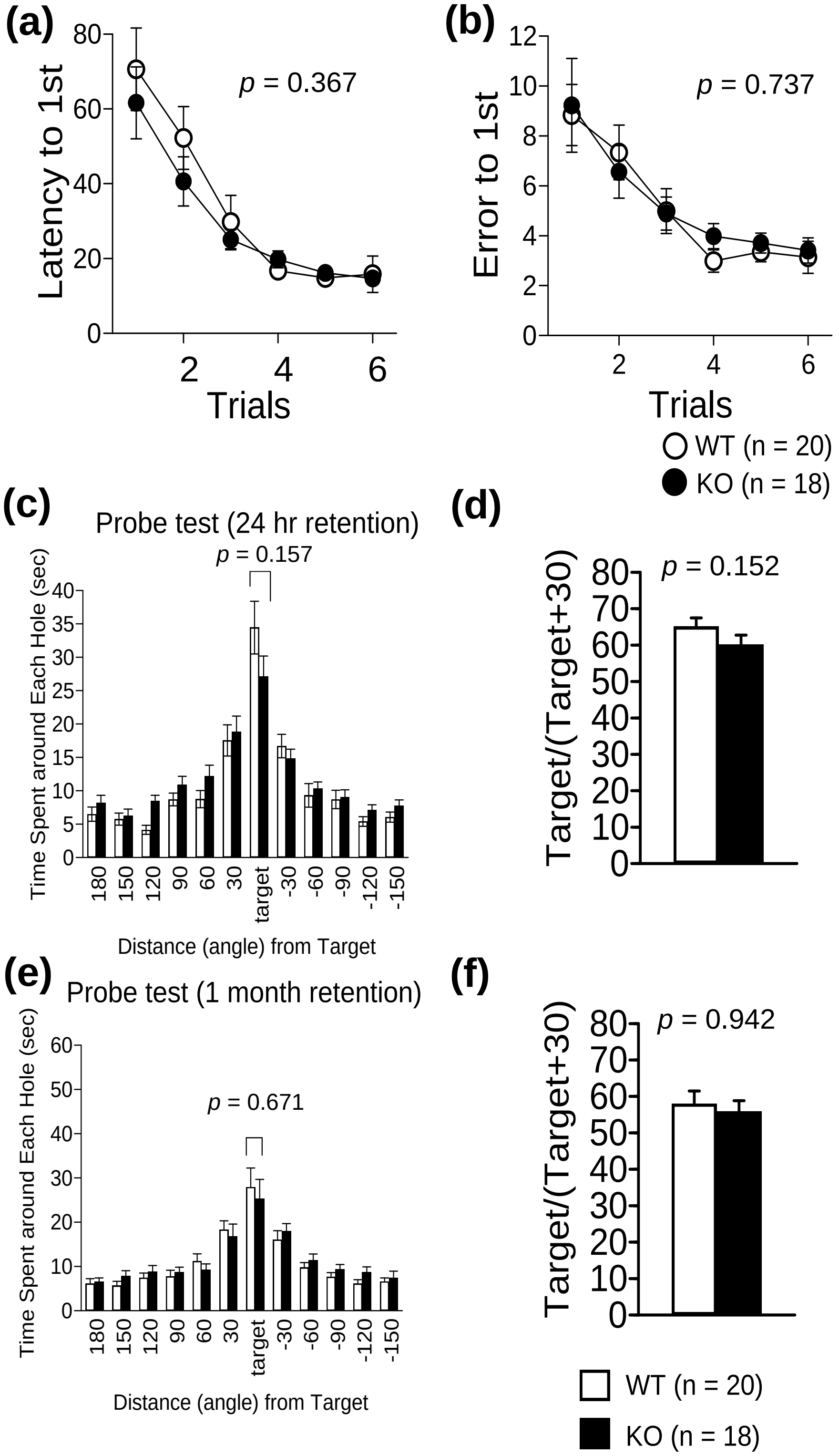

Supplemental Figure S6: Barnes maze test. (a, b) Training course: Latency to the target hole (a) and the number of errors to the target hole (b) were recorded. (c, d) Probe tests 24 hours after the last training: Time spent around each hole (c) and the ratio of time spent around the target and target + ±30 (d) were recorded. (e, f) Probe tests 1 month after the last training: time spent around each hole (e), ratio of time spent around the target and target + ±30 (f) were recorded. The *p*-values indicate a genotype effect in the two-way repeated measures ANOVA (a, b), one-way ANOVA (c, e) and Mann-Whitney *U*-test (d, f). Data are given as mean (±SEM).
