# Supplementary material for: Comprehensive behavioral study of mGluR3 knockout mice: implication in schizophrenia related endophenotypes
Source: Mol Brain. 2014 Apr 23;7:31. doi: 10.1186/1756-6606-7-31 (PMC4021612; doi:10.1186/1756-6606-7-31)
Supplement: Additional file 7: Figure S7 — Passive avoidance test. (a, b) Latency to enter dark compartment after one day (a) and two days (b) were recorded. The p-values indicate a genotype effect in the one-way ANOVA. Data are given as mean (±SEM). [file 1756-6606-7-31-S7.pdf]

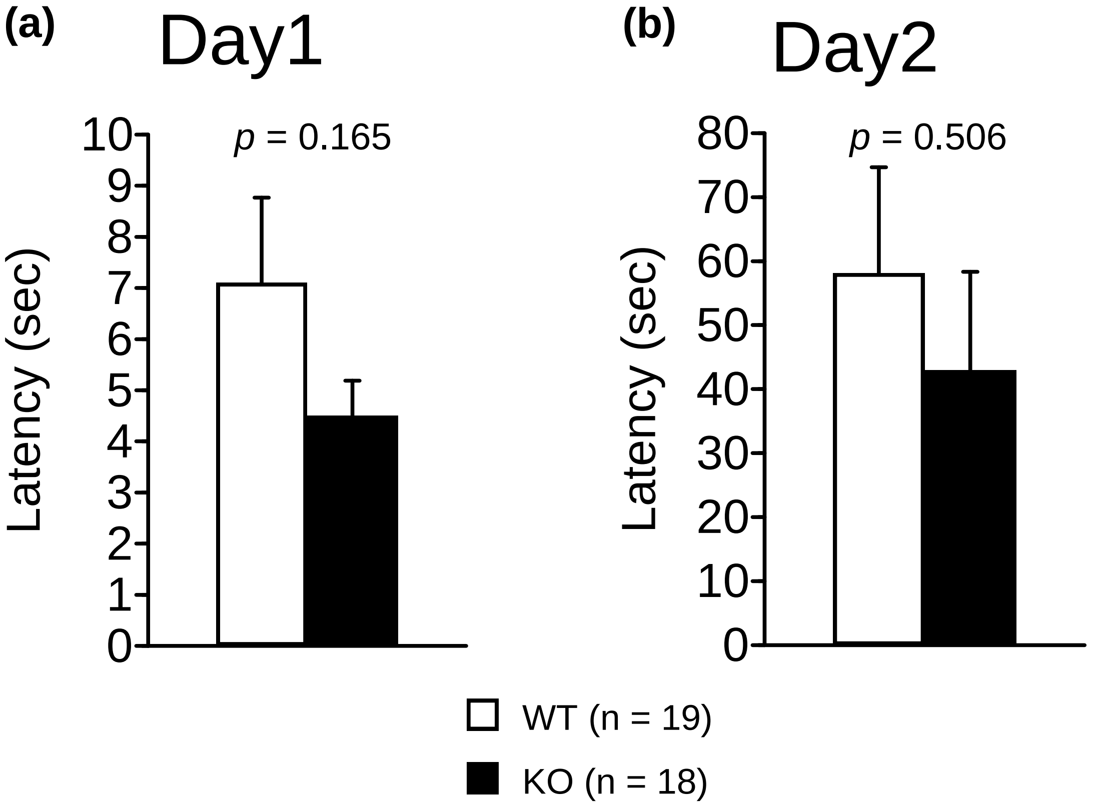

Supplemental Figure S7: Passive avoidance test. (a, b) Latency to enter dark compartment after one day (a) and two days (b) were recorded. The *p*-values indicate a genotype effect in the one-way ANOVA. Data are given as mean (±SEM).
